# Japanese Interest in “Hotaru” (Fireflies) and “Kabuto-Mushi” (Japanese Rhinoceros Beetles) Corresponds with Seasonality in Visible Abundance

**DOI:** 10.3390/insects3020424

**Published:** 2012-04-10

**Authors:** Kenta Takada

**Affiliations:** 3-13-29, Takejima, Nishiyodogawa-ku, Osaka, 555-0011, Japan; E-Mail: athemus99@yahoo.co.jp; Tel.: +81-6-6473-4128; Fax: +81-6-6473-4128

**Keywords:** fireflies, Japanese rhinoceros beetles, seasonal interest, the Japanese, popularity, Google Trends, cultural entomology

## Abstract

Seasonal changes in the popularity of fireflies [usually Genji-fireflies (*Luciola cruciata* Motschulsky) in Japan] and Japanese rhinoceros beetles [*Allomyrina dichotoma* (Linne)] were investigated to examine whether contemporary Japanese are interested in visible emergence of these insects as seasonal events. The popularity of fireflies and Japanese rhinoceros beetles was assessed by the Google search volume of their Japanese names, “Hotaru” and “Kabuto-mushi” in Japanese Katakana script using Google Trends. The search volume index for fireflies and Japanese rhinoceros beetles was distributed across seasons with a clear peak in only particular times of each year from 2004 to 2011. In addition, the seasonal peak of popularity for fireflies occurred at the beginning of June, whereas that for Japanese rhinoceros beetles occurred from the middle of July to the beginning of August. Thus seasonal peak of each species coincided with the peak period of the emergence of each adult stage. These findings indicated that the Japanese are interested in these insects primarily during the time when the two species are most visibly abundant. Although untested, this could suggest that fireflies and Japanese rhinoceros beetles are perceived by the general public as indicators or symbols of summer in Japan.

## 1. Introduction

The field of cultural entomology examines the influence of insects on human culture [1,2,3,4,5,6] and explores which and how insects are used and perceived in human societies. In the field of cultural entomology, Japan has been mentioned, because Japanese traditionally appreciate insects for their beauty and vulnerability [1,3,7,8]. Indeed, certain species are being used in a variety of ways and meanings in Japanese society [8,9,10].

In contemporary Japan, fireflies (especially the common species of Genji-fireflies: *Luciola cruciata* Motschulsky) and Japanese rhinoceros beetles [*Allomyrina dichotoma* (Linne)] have strongly fascinated the general public, with the result that they are some of the most popular insects [4,11], and have assumed a position of special cultural significance in Japanese culture [3,4,5,8,12,13]; therefore, it is meaningful to analyze how these insects are used and perceived in Japanese culture.

In particular, in Japan, adult emergences of fireflies and Japanese rhinoceros beetles have been perceived as familiar seasonal events of early and mid-summer for many peoples, respectively [8,12,13,14]. In fact, some authors have reported that haiku, which is a very short form of Japanese poetry with seasonal references, traditionally praises such insects as synonymous of the summer season [7,12,13,15,16]. However, it has still not been well examined whether contemporary Japanese are interested in insects primarily during the time when the two species are most visibly abundant because trends in the popularity of insects across seasons have never been assessed quantitatively. 

Trends in the popularity of fireflies and Japanese rhinoceros beetles were therefore investigated across seasons to examine whether these insects interest the Japanese in correspondence with their seasonal abundance. The interest of the Japanese in visible emergence of these insects as seasonal events is indicated by their popularity across seasons with a clear peak in only particular times of the year. In addition, the seasonal peak of popularity for each insect occurs in early and mid-summer, respectively. The popularity of fireflies and Japanese rhinoceros beetles was assessed by the Google search volume of their Japanese names, “Hotaru” and “Kabuto-mushi”. The search volume is used as a yardstick to measure a term’s intention, interest or popularity [17,18,19], and thus can be applied for investigating the popularity of insects [4,20] as well as internet marketing and search engine optimization [17,18,19]. Although this analysis technique is inapplicable for less popular insects, whose names are not frequently searched using Google, the high popularity of these insects allowed the analysis of the trend in their popularity across seasons. 

## 2. Experimental Section

### 2.1. Materials

**Figure 1:**
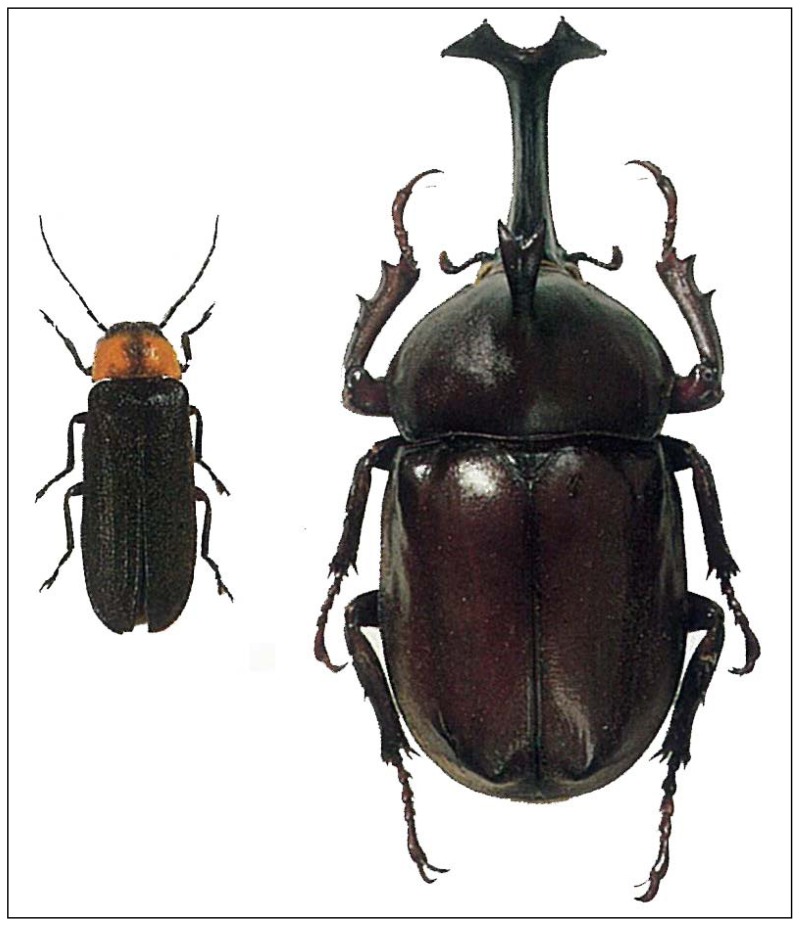
The common lampyrid species of Genji-fireflies (*Luciola cruciata* Motschulsky) (10–16 mm in length) and male of Japanese rhinoceros beetle [*Allomyrina dichotoma* (Linne)] (32–53 mm in length). These figures are cited from Sato [21] and Kurosawa *et al*. [22], respectively (in The Coleoptera of Japan in Color published by Hoikusha).

This study focused on fireflies (Lampyrids) and Japanese rhinoceros beetles [*Allomyrina dichotoma* (Linne)] (Figure 1). In Japan, fireflies are called “Hotaru”, which is the familial and common lampyrid name but usually refers to Genji-fireflies (*Luciola cruciata* Motschulsky) [20] occurring in early summer (from May until July) [21]. On the other hand, Japanese rhinoceros beetles [*Allomyrina dichotoma* (Linne)] are called “Kabuto-mushi” and occur in mid-summer (from June until August) in Japan [22]. These insects are distributed in rural areas over a wide range of the Japanese main islands [21,22].

### 2.2. Method

A survey was conducted on seasonal changes in the popularity of fireflies and Japanese rhinoceros beetles on 11 November 2011, assessing the search volume over time using Google Trends (http://www.google.co.jp/trends). Google Trends analyzes a portion of Google web searches to compute how many searches have been performed for the terms entered, relative to the total number of searches performed on Google over time. There are two modes of scaling “relative and fixed” in Google Trends, and to measure the search volume index, the relative mode of scaling is used in Google Trends. In relative mode, the data are scaled to the average search traffic for the term (represented as 1.0) during the time period selected in contrast to fixed mode, in which the data are scaled to the average traffic for your term during a fixed point in time (usually January 2004).

The search volume index of Japanese names for fireflies (“Hotaru”) and Japanese rhinoceros beetles (“Kabuto-mushi”) was assessed in katakana, a Japanese syllabic script used in Japanese writing system (Table 1). Katakana is most often used for the transcription of words from foreign languages, onomatopoeia and technical and scientific terms, such as the names of animal and plant species and minerals [4,20]. Thus Katakana is more suitable than other Japanese scripts such as Hiragana and Kanji to examine which and how insects are used and perceived in Japanese culture. The search volume index of butterflies (“Chou”) was also assessed in katakana as a control term for the sake of comparison. In Japan, butterflies are popular insects and are synonymous with Spring (but sometimes with other seasons) in Haiku poetry. Butterflies, in contrast to the two beetle species, are found as adults from spring until autumn.

**Table 1:** Japanese transcription (Katakana) of the firefly (Lampyridae) and Japanese rhinoceros beetle (*Allomyrina dichotoma* (Linne)).

English	Japanese reading	Japanese writing(Katakana scripts)
firefly	Hotaru	ホタル
Japanese rhinoceros beetle	Kabuto-mushi	カブトムシ

Mozilla Firefox 3.6.24 was used to evaluate the search volume The operating system was Windows XP Version 2002 Service Pack 3 installed on a FUJITSU Esprimo D760/AX (CPU: Intel Core i5 CPU650 (3.2 GHz)).

## 3. Results and Discussion

**Figure 2:**
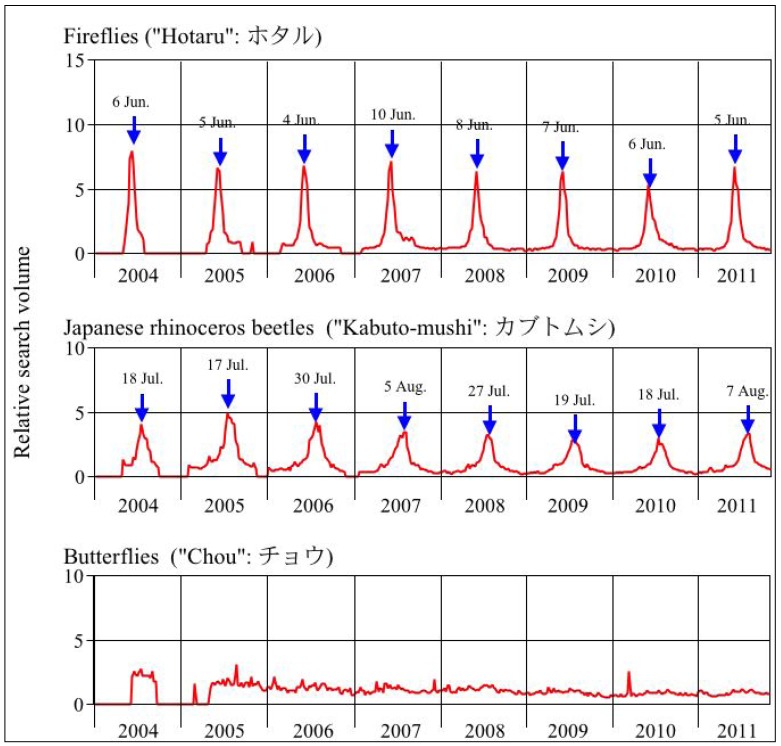
Seasonal change in relative search volume index for “Hotaru” (fireflies) and “Kabuto-mushi” (Japanese rhinoceros beetles) Relative search volume index for the control term “Chou” (butterflies) is plotted over time.

The search volume index for fireflies and Japanese rhinoceros beetles was distributed across seasons with a clear peak at only particular times of each year, in contrast with butterflies whose search volume index was almost constant across seasons from 2004 to 2011 (Figure 2). In addition, the seasonal peak of popularity for fireflies occurred at the beginning of June, whereas that for Japanese rhinoceros beetles occurred from the middle of July to the beginning of August. These results are considered reliable indicators of the insects’ popularity across seasons because of the high abundance of computers in Japan (76% of total number of households) [23], although internet use is somewhat biased in favor of younger and well-educated people [17]. For each insect, the seasonal peak in the search volume index mostly coincided with the peak period of the occurrence of adult stage. These findings indicated that the Japanese are interested in these two insects mostly during early and mid-summer, respectively, suggesting that fireflies and Japanese rhinoceros beetles could be perceived as indicators or symbols of summer. Although this claim remains to be tested, several studies make it clear that these insects give poetic, seasonal charm to Japanese culture [8,12,13].

The meteorological sensitivity of fireflies and Japanese rhinoceros beetles allows them to react to seasonal atmospheric variations and indicate a particular season for people. In both insects, seasonal occurrences are restricted to a particular short season of the year with periodic change in atmospheric variations. Moreover, (1) their conspicuous biological traits, such as bioluminescence produced by swarms of fireflies, and the large body and distinctive long horn of Japanese rhinoceros beetles, (2) their occurrence around human habitation (but in rural landscapes) and (3) the widespread distribution of the species on the main islands of Japan, enables people to notice these insects.

The Japanese are interested in fireflies and Japanese rhinoceros beetles as subjects of seasonal pastimes, and the insects carry a positive image. Neither insect is used for practical purposes such as food and medicine, and is not considered as a pest with a negative image, but is used for the appreciation of nature or reared as a pet [5,12,13,14]. For example, historically, chasing these lampyrid species for their bioluminescence (*Hotaru-gari*) has been a traditional pastime during early summer evenings in Japan [3,4,5,13,14,24,25,26,27]. In recent Japan, Japanese rhinoceros beetles have strongly fascinated the general public, with the result that they are popular as pets and thus have a large market, and rearing Japanese rhinoceros beetles has been a popular activity for Japanese during mid-summer [12,14]. 

It is probable that the Japanese seasonal interest in fireflies and Japanese rhinoceros beetles stems from these species’ esthetic value during times when the land is rich in resources used by humans. Adults of these insects occur in the Japanese traditional agricultural region called “Satoyama”, which consists of a mosaic of forests, grasslands, rice paddy fields, ponds, creaks and irrigation ditches that have historically provided resources for agricultural life [28], and emerge during the agricultural growing season, and thus may be associated with a resource-rich environment and a good season for many people.

In addition, the Japanese seasonal interest in these insects may also be related to the Japanese fundamental and essential concept of being Japanese, called “Mono no Aware”, which characterizes beauty as the transience of all things and defines true beauty as being found in that which does not last and includes the gentle sadness felt as it fades [16]. In fact, the Japanese often represent a spontaneous moment of the transient things of nature, such as insect life and flowering plants in haiku, which often praises insects (including fireflies and Japanese rhinoceros beetles) as synonymous of particular season, bringing a moment’s smallest details of a particular time and space into focus with a small number of words [7,15,16].

## 4. Conclusions

Japanese are interested in fireflies and Japanese rhinoceros beetles primarily during the time when the two species are most visibly abundant. Although untested, this could suggest that fireflies and Japanese rhinoceros beetles are perceived by the general public as indicators or symbols of summer in Japan. The Japanese seasonal interest in these insects might stem from these species’ esthetic value during resource-rich seasons, and may be related to the Japanese fundamental and essential concept of being Japanese, called “Mono no Aware”, which characterizes beauty as the transience of all things. Moreover, the two species harmonize with the natural feature of Japan with its clear periodic seasonal changes and the Japanese disposition towards an appreciation of insect life.
